# Thermophilic endospores associated with migrated thermogenic hydrocarbons in deep Gulf of Mexico marine sediments

**DOI:** 10.1038/s41396-018-0108-y

**Published:** 2018-03-29

**Authors:** Anirban Chakraborty, Emily Ellefson, Carmen Li, Daniel Gittins, James M. Brooks, Bernie B. Bernard, Casey R. J. Hubert

**Affiliations:** 1 Department of Biological Sciences, University of Calgary, Calgary, AB Canada; 2 TDI Brooks International, College Station, TX USA

## Abstract

Dormant endospores of thermophilic bacteria (thermospores) can be detected in cold marine sediments following high-temperature incubation. Thermospores in the cold seabed may be explained by a dispersal history originating in deep biosphere oil reservoir habitats where upward migration of petroleum fluids at hydrocarbon seeps transports viable cells into the overlying ocean. We assessed this deep-to-shallow dispersal hypothesis through geochemical and microbiological analyses of 111 marine sediments from the deep water Eastern Gulf of Mexico. GC-MS and fluorescence confirmed the unambiguous presence of thermogenic hydrocarbons in 71 of these locations, indicating seepage from deeply sourced petroleum in the subsurface. Heating each sediment to 50 °C followed by 16S rRNA gene sequencing revealed several thermospores with a cosmopolitan distribution throughout the study area, as well as thermospores that were more geographically restricted. Among the thermospores having a more limited distribution, 12 OTUs from eight different lineages were repeatedly detected in sediments containing thermogenic hydrocarbons. A subset of these were significantly correlated with hydrocarbons (*p* < 0.05) and most closely related to *Clostridiales* previously detected in oil reservoirs from around the world. This provides evidence of bacteria in the ocean being dispersed out of oil reservoirs, and suggests that specific thermospores may be used as model organisms for studying warm-to-cold transmigration in the deep sea.

## Introduction

A large proportion of Earth’s biomass, estimated to be close to 10^30^ cells, is found in dark warm subsurface habitats where biodiversity is comprised exclusively of microorganisms [1–5]. Recent research has shown active populations of anaerobic microbes, many of which are thermophiles, in deeply buried anoxic sediments, oil reservoirs [6, 7], permeable ocean crust [8], and around hydrothermal vents at mid-ocean ridges [9]. Geological features enabling seabed fluid flow from warm to cold environments, such as hydrocarbon seeps and mid-ocean ridge-associated vents or seamounts, physically connect subsurface ecosystems to the overlying oceans [10, 11]. The transport of cells from subsurface habitats up into the overlying ocean may contribute to marine microbial biodiversity [12, 13], including the well documented ‘rare biosphere’ [14–17]. This marine microbial seed bank is largely comprised of dormant microorganisms [18], including inactive endospores of thermophilic bacteria, i.e., thermospores [19–21].

Microbial biodiversity surveys generally do not detect bacterial endospores, most likely due to endospore resistance to physical or chemical lysis steps employed during community DNA extraction from environmental samples [22, 23]. High temperature, anoxic incubations have therefore been employed to study thermospores [24] and have revealed annual influx rates of 10^7^ and 10^8^ spores per square meter in the cold seabed sediments of Aarhus Bay and Svalbard, respectively [19, 25]. These thermospores include close relatives of anaerobic *Clostridia* that inhabit hot petroleum reservoirs [26–28]. A large-scale investigation of thermospore biogeography from 81 marine sediments showed a non-random distribution pattern globally and identified ocean circulation as a major contributor in controlling thermospore dispersal from their source habitats [20]. Taken together, high local influx rates, phylogenetic similarity with oil-reservoir-derived microorganisms, and non-random distribution suggest a dispersal history of thermospores influenced by vectors facilitating passive transport from petroleum-bearing subsurface strata characterized by sufficient magnitude and fluid flow to maintain a steady supply of endospores [29]. Seabed hydrocarbon seeps, where gas and oil in leaky subsurface reservoirs are expelled up to the seafloor, offer an example of an active geofluid-flow system linking the deep and shallow biospheres [11] that could satisfy the above criteria for thermospore dispersal.

Marine sediments in and around hydrocarbon seeps are generally replete with petroleum-associated compounds and provide access to plethora of organic growth substrates for microbial communities compared to otherwise oligotrophic deep sea sediments [30]. Hydrocarbon seeps thus host microbiomes which are locally selected and are largely distinct from surrounding seafloor ecosystems [31]. The Gulf of Mexico (GoM) basin is well known for widespread natural seepage of petroleum-derived hydrocarbons sourced from deeply buried oil and gas reservoirs, with an estimated annual oil discharge rate of more than 30,000 m^3^ [32–34]. Cold-temperature-adapted microbial communities at GoM seeps have thus been studied in great detail in relation to the impact of hydrocarbons on community diversity [35, 36], sulfate reduction [37], and hydrocarbon biodegradation [38] whereas thermospores have received far less attention. Given that endospore-forming anaerobic *Clostridia* inhabit hot petroleum reservoirs such as those in the GoM basin, it is conceivable that these endospores could migrate upward with naturally seeping petroleum fluids and eventually get deposited in the overlying surface sediments [29]. Consistent with this, recent investigations of six reservoirs in the adjacent (onshore) Gulf coastal plain revealed a predominance of *Clostridiales* [39] adding to the number of studies indicating that thermophilic *Firmicutes* are prevalent in high-temperature oil fields [40].

We therefore addressed the following questions in this study; are thermospores present in petroleum-rich surface sediments of the Gulf of Mexico? Can regional-scale biogeography of thermospores reveal distinguishable distributions in marine sediments with geochemical signatures of migrated thermogenic hydrocarbons? Can hydrocarbon seep-driven endemism of thermospores be observed, i.e., do anomalies for taxa and hydrocarbons co-exist? To address the above questions, we analyzed the phylogeny and distribution of thermospores in 111 marine sediments from the Eastern Gulf of Mexico (EGoM) while simultaneously assessing thermogenic hydrocarbon content by measuring a suite of geochemical parameters. Previous investigations of thermospores predicted but could not directly demonstrate deep subsurface origins of these organisms. Our study addresses this by investigating thermospores in marine sediments with direct evidence of migrated hydrocarbons.

## Materials and methods

### Sampling of marine sediments

**Fig. 1:**
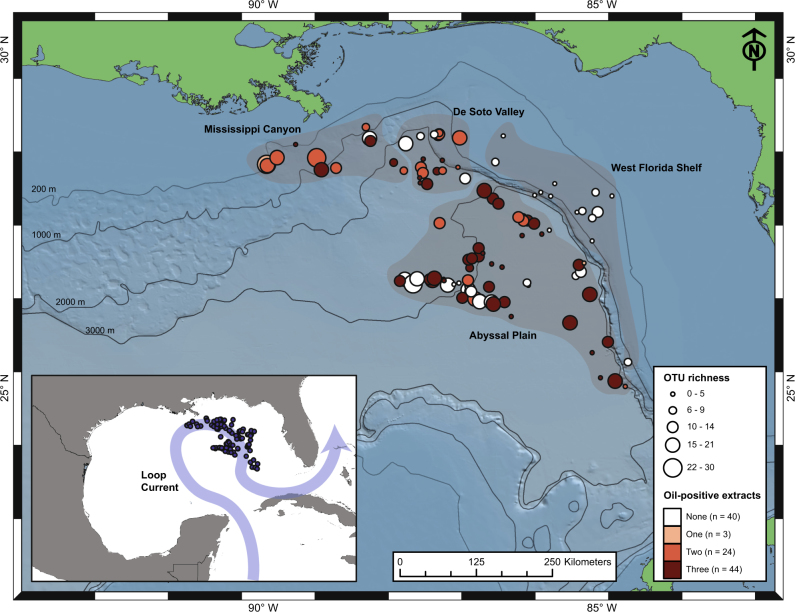
Map of the Eastern Gulf of Mexico (GoM) showing the 111 sampling locations and bathymetry of the study area. Circle sizes indicate thermospore OTU richness for each location. Color shades of the circles indicate the number of oil-qualified extracts (*n* = 0, 1, 2, 3) in the sediment core for that location. The semi-transparent dark polygons indicate four different geologic provinces within the sampling area. The inset map shows the location and extent of the study area relative to the area of the entire GoM basin as well as the region affected by the Loop Current, the major ocean current in the GoM. Maps were drawn using ArcGIS Desktop 10.4

Marine surface sediments (0–20 cm below seafloor) were collected from 111 locations in the Eastern Gulf of Mexico (Fig. 1; Supplementary Table S1) during January–March, 2011, aboard RV GeoExplorer as part of TDI Brooks International’s Surface Geochemical Exploration (SGE) program. Sediments were sampled from a range of water depths, and were distributed within four geologic provinces: the Abyssal Plain (*n* = 66; average water depth = 3024 m), the De Soto Valley (*n* = 19; average water depth = 2143 m), the Mississippi Canyon (*n* = 12; average water depth = 982 m), and the West Florida Shelf (*n* = 14; average water depth = 942 m). The overall average water depth was 2390 m with a large majority (*n* = 82/111) being >2000 m. Pairwise geographic distances between sampling locations ranged from 0.23 to 640 km. Piston cores penetrating 4.5–5.5 m depth were collected and immediately extruded into 20 cm sections. Surface sediments for microbiological analyses were sealed in sterile Whirl-Pak bags with minimum air exposure and kept frozen at −20 °C. Deeper intervals for thermogenic hydrocarbon analyses were also frozen at −20 °C. For analysis of interstitial light hydrocarbon gases, a portion of the deepest section was placed in 500 ml gas cannisters containing clean, degassed, and sterilized seawater. Cannisters were purged with nitrogen and stored at −20 °C.

### **Hydrocarbon characterization**

Hydrocarbon analyses were performed on solvent extracts prepared from three equidistant segments from the bottom half of each piston core, representing a depth range of 2–5.5 m below seafloor. Frozen sediments were thawed, oven-dried at 40 °C, and 15 g portions were solvent extracted with hexane using an automated extraction apparatus (Dionex ASE200). Liquid petroleum hydrocarbons were analyzed using (1) total scanning fluorescence (TSF) intensities, a semi-quantitative analytical technique selectively sensitive to aromatic hydrocarbons, and (2) gas chromatography of the same extracts to obtain total concentrations of C_15_+ hydrocarbons, including determination of the unresolved complex mixture (UCM). A detailed description of the above geochemical methods has been provided elsewhere [41].

Hydrocarbon parameters are presented as the mean of individual measurements obtained from three extracts per piston core described above. To group locations based on the above measurements, thresholds were set for TSF and UCM. A conventional TSF maximum intensity threshold of 10,000 is often used as an indicator of migrated petroleum in prospective basins around the world [41]; however, this threshold was elevated five-fold (50,000 intensity units) for interpreting the TSF values obtained in this study. This much more stringent TSF threshold was chosen to account for the relatively large input of terrigenous organic matter in the GoM compared to other basins [42]. If an extract or a location gave individual or mean TSF intensities of less than 50,000 and a UCM of lower than 10 µg/g sediment, the extract or the location was disqualified for unambiguous occurrence of thermogenic hydrocarbons. Based on these thresholds, the locations were classified as ‘oil-negative’ and ‘oil-positive’, respectively.

### Incubation of pasteurized sediment slurries at 50 °C

Triplicate slurries were prepared from 0 to 20 cm surface sediment from each of the 111 locations in order to investigate thermospore germination and growth. Non-homogenized, frozen sediments were thawed overnight and three separate aliquots (approximately 10 g each) were directly added to three separate sterile 50 ml serum bottles inside a walk-in cold room (4 °C). Serum bottles were immediately sealed with sterile black butyl stoppers and the headspace exchanged with N_2_:CO_2_. Sediment aliquots were subsequently diluted in a 1:2 (w/w) ratio with anoxic, artificial seawater medium [43] containing 20 mM sulfate under a constant flow of N_2_:CO_2_. To minimize competition for limited substrates between different microbial groups (e.g., sulfate-reducing and fermentative bacteria) the slurries were amended with a combination of six low-molecular-weight organic acids (acetate, butyrate, formate, lactate, propionate, and succinate), each at a final concentration of 5 mM. Slurries were pasteurized at 80 °C for 1 h to eliminate viable vegetative cells and were incubated at 50 °C immediately afterwards to promote germination and growth of thermophilic endospores. Initial time-zero samples were taken before pasteurization and slurries were incubated for 2 weeks, with sub-sampling every 2–3 days. Subsamples (1–2 ml) were removed using N_2_:CO_2_-flushed sterile syringes and immediately centrifuged at 14,000 × *g* for 10 min. Resulting supernatant and pellet samples were stored separately at −20 °C and used for liquid chromatography (see Supplementary Methods) and DNA extractions, respectively.

### **16S rRNA gene amplicon sequencing**

Genomic DNA was extracted from subsamples of triplicate sediment slurries after 2 weeks of incubation (14-day), and from the corresponding unheated in situ sediments (0-day). Equal volumes of slurry from each replicate subsample were pooled and the mixed slurry was used for DNA extraction using the PowerLyzer PowerSoil DNA isolation kit (now known as the DNeasy PowerLyzer PowerSoil kit, MO BIO Laboratories, a Qiagen Company, Carlsbad, CA, USA). The v3-4 region of the bacterial 16S rRNA gene was amplified using the primer pair SD-Bact-341-bS17/SD-Bact-785-aA21 [44] modified with Illumina MiSeq overhang adapters. All PCR reactions were performed in triplicate, pooled, purified, and indexed according to Illumina’s 16S amplicon library preparation guide (see Supplementary Methods for more details). Indexed amplicons were then pooled in equimolar amounts and sequenced using Illumina’s v3 600-cycle (paired-end) reagent kit on a MiSeq benchtop sequencer (Illumina Inc., San Diego, CA, USA). Bacterial diversity in 0-day sediment samples was anticipated to be greater than in 14-day sediment samples, so these samples were loaded onto the MiSeq sequencer at twice the DNA concentration to ensure at least 10,000 and 5000 reads for 0-day and 14-day libraries, respectively, post quality control.

### Sequence processing and diversity analyses

In total, 13,662,481 paired-end raw reads were processed using the MetaAmp web-based bioinformatics pipeline [45]. After assembly, quality control, de-replication, removal of singletons, and chimeric sequences, 5,119,876 usable single reads were clustered into operational taxonomic units (OTUs) based on a 97% sequence identity threshold over 350 bases. All libraries were rarefied to 5000 reads in order to eliminate biases arising from variability in library size. Alpha-diversity metrics (observed OTU richness, chao1 richness, Shannon and Simpson diversity indices, and equitability index) were calculated from the rarefied libraries using Mothur version 1.35.1. For beta diversity analyses, the libraries were grouped separately based on before and after incubation (0-day and 14-day). Community similarities were measured using the Bray-Curtis and weighted UniFrac indices. All beta diversity analyses were conducted using the phyloseq R package [46]. Amplicon sequences generated in this study are available through the NCBI Sequence Read Archive (BioProject accession number PRJNA415828).

### Identification of putative thermospore OTUs

Putative thermospore OTUs were defined as follows. First, OTUs had to be affiliated with the phylum *Firmicutes*, to which all known endospore-forming bacteria belong [47]. Second, each OTU had to significantly increase in relative abundance in at least one heated sediment incubation; this is based on the assumption that many thermospores will germinate and grow at 50 °C after surviving the initial pasteurization. Significant increase of an OTU was defined as a 0.5% increase in relative abundance in a 14-day library (i.e., 25 sequences), and validated by performing a two-proportion *Z*-test between the 0- and 14-day libraries from the same locations. Differences of proportions amounting to 0.5% relative abundance and higher corresponded to *p*-values of 0.0001 and lower, and were considered significant. Subsequently, each thermospore OTU was evaluated in all other sediments and considered present if its relative abundance was at least 0.1% (5 sequences) in 14-day rarefied amplicon libraries for other locations. These criteria were applied to avoid biases from cross-sample or carry-over contamination during sequencing on the MiSeq platform [45, 48]. Based on these criteria, a presence–absence matrix for thermospore OTUs was calculated and visualized as bipartite networks showing the OTU connectivity with sampling locations using the igraph R package [49]. The association of each OTU with the parameters indicative of migrated hydrocarbons (TSF and UCM values) was evaluated using point biserial correlation (R package “wCorr”) [50] and the correlation coefficients (*r*_pb-TSF_ and *r*_pb-UCM_) were reported for OTUs showing significant correlation.

### Phylogeny

Representative sequences from MetaAmp for thermospore OTUs that significantly increased in relative abundance were automatically aligned using the web-based SINA aligner [51] and imported into the ARB-SILVA database SSU Ref NR 128 [52] using the ARB software package [53]. A maximum likelihood (PhyML) tree was calculated using almost full-length 16S rRNA sequences (1400 nt) from closely related reference bacteria or environmental clones based on 1183 alignment positions by using positional variability and termini filters for bacteria. Using the ARB Parsimony tool, the short amplicon sequences were added to this tree applying the 50% sequence conservation filter and positional variability filters covering the length of the representative sequences for each OTU without changing the overall tree topology. Trees were visualized and annotated using iTOL version 3 [54].

## Results

### Migrated hydrocarbons in deep water Gulf of Mexico surface sediments

**Fig. 2:**
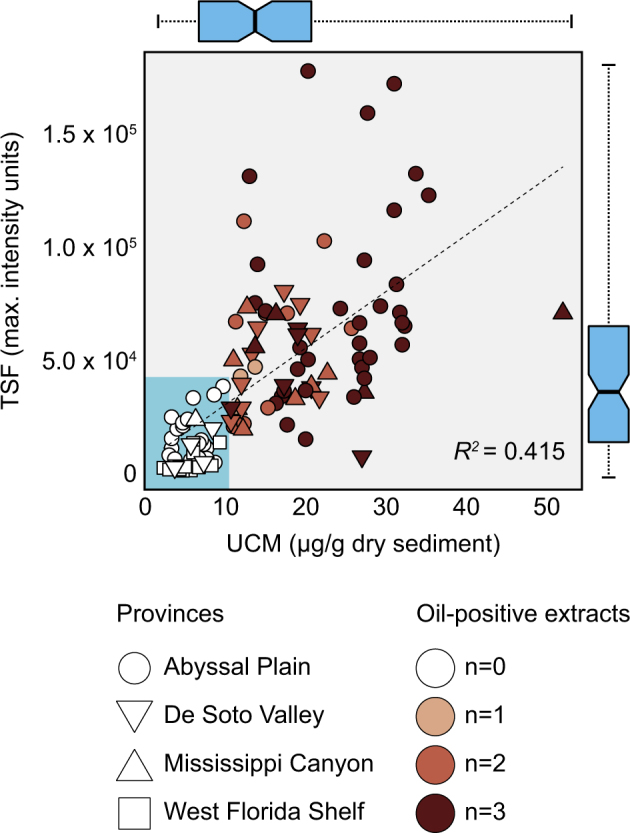
Scatterplot showing the mean values (*n* = 3 extracts) of two geochemical parameters used for assessing the thermogenic hydrocarbon content in 111 sediment cores. The plot is accompanied by two marginal box-and-whisker plots summarizing the distribution (minimum, lower quartile, median, upper quartile, and maximum) for each of the parameters on the corresponding parallel axes. The dashed line represents the linear regression of the parameters with the corresponding *R*^*2*^ value indicated. The cyan-shaded box highlights the UCM and the TSF (maximum intensity) thresholds used by industry for qualifying and/or disqualifying individual sediment extracts for thermogenic hydrocarbon content. Symbol colors indicate the number of qualified extracts (*n* = 0, 1, 2, or 3 out of 3) in each sediment core, according to the thresholds. Symbol shapes indicate the different geologic provinces

The distributions of migrated hydrocarbons and thermophilic endospores were investigated in four geological provinces in the Eastern Gulf of Mexico (Fig. 1). TSF intensities and the mass of the UCM were used as the two major indicators of migrated liquid petroleum, since the ‘thermogenicity trend’ of these two parameters remains linear over several orders of magnitude [55, 56]. TSF intensities are generally independent of microbial alteration of hydrocarbon compounds and provide a measure of petroleum-related aromatic hydrocarbon concentrations. The UCM on the other hand represents a suite of biodegraded saturated hydrocarbons that are not easily separated using gas chromatography, resulting in a hump-shaped baseline with numerous smaller peaks representing individual compounds. TSF and UCM values ranged from 1800 to 178,016 intensity units and 2 to 52 µg/g dry sediment, respectively. These two parameters were positively correlated (*R*^*2*^ = 0.415; *n* = 111) among all locations (Fig. 2). The thermogenic nature of the hydrocarbons was further confirmed through measurement of total C_15+_ alkanes and the thermogenic/diagenetic (T/D) ratio (see Supplementary Results and Supplementary Figure S1 for more details).

To evaluate thermospore distribution in accordance with the presence of migrated petroleum, thresholds were set for TSF and UCM. Based on these thresholds, 40 and 71 locations were classified as oil-negative and oil-positive, respectively (Fig. 2, Supplementary Table S1). Among the oil-positive locations, 44 had very strong indications, with all 3 sediment extracts from the same piston core being above TSF and UCM thresholds, while 24 had two-out-of-three qualified extracts, and 3 locations had only one qualified extract (Figs. 1 and 2). The Mississippi Canyon province contained the highest percentage of oil-positive samples (91.7%; *n* = 11/12), while all 14 samples from West Florida Shelf were oil-negative (Fig. 1). In addition to having low TSF and UCM values, sediments from the 14 relatively shallow West Florida Shelf locations showed very low values for other hydrocarbon parameters while the average concentration of C_15+_ alkanes was highest (2.5 µg/g dry sediment) in the Mississippi Canyon province (Supplementary Figure S1).

### Diversity of thermophilic endospores in deep water Gulf of Mexico surface sediments

**Fig. 3:**
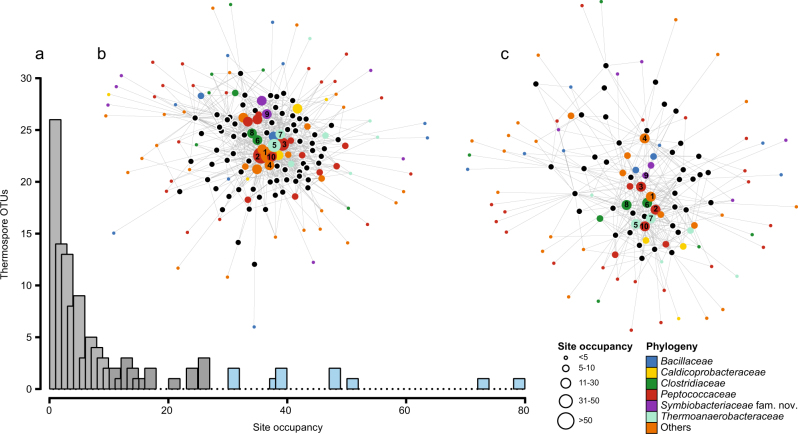
**a** Histogram showing the site occupancy of the thermophilic endospore OTUs. The *X* and *Y* axes represent the number of locations at which each OTU was detected and the number of OTUs detected at that frequency, respectively. The light blue bars represent the top ten cosmopolitan OTUs found in >30 locations. **b**, **c** Bipartite network graphs of (**b**) oil-positive (*n* = 70) and (**c**) oil-negative (*n* = 40) sampling locations based on occurrence of thermophilic endospore OTUs. Colored nodes represent OTUs present in **b** oil-positive (*n* = 102 OTUs) and **c** oil-negative (*n* = 85 OTUs) locations. The colors of the OTU nodes indicate Family level taxonomy and the circle sizes indicate their site occupancy. Black nodes represent the sampling locations to which the gray lines connect the OTUs. The distance between the black location nodes reflects their thermospore OTU connectivity, e.g., locations sharing many OTUs are plotted close to each other in the inner periphery of the network whereas those that share few OTUs plot distantly to each other in the outer periphery. The top ten cosmopolitan OTUs are numbered on both networks and are plotted in the middle, while endemic and site-specific OTUs are plotted on the periphery

Investigating various incubation conditions on a subset of pasteurized EGoM sediments (see Supplementary Results for more details) revealed that the maximum number of thermospores were detected in slurries amended with a mix of six organic acids after 14 days of incubation at 50 °C (Supplementary Figure S2) with comparable diversity within the *Firmicutes* observed among triplicate slurries (Supplementary Figure S3). Anoxic incubations in the above conditions of all 111 EGoM sediments generally resulted in reduced bacterial alpha diversity (Supplementary Table S2). Principal coordinate analysis of weighted UniFrac distances and non-metric multidimensional scaling analysis of Bray-Curtis distances both confirmed a shift in community structure in terms of phylogenetic composition of bacterial 16S rRNA genes and OTU counts, respectively, both before (0-day) and after (14-day) pasteurization and 50 °C incubation in most but not all samples (Supplementary Figure S4). *Firmicutes* was, as expected, the dominant phylum in almost all of the 14-day libraries (*n* = 110/111) representing 92% of total sequences and with relative abundances ranging from 35 to 99.9% across individual libraries. The fraction of total OTUs affiliated to *Firmicutes* increased from 3.2% (*n* = 143/4490) in the rarefied 0-day libraries to 32.5% (*n* = 314/967). Subsequently, a total of 115 thermospore OTUs were identified across all high-temperature incubations (Supplementary Table S3; Supplementary Figure S5). None of these OTUs were detected in any of the 0-day amplicon libraries (before pasteurization and incubation), consistent with these organisms being present in situ as endospores that germinated upon exposure to the high incubation temperature. Thermospore OTUs were detected in samples from all of the investigated locations except one (Fig. 1). Most of these OTUs were affiliated with the orders *Clostridiales* (62.6%, *n* = 72), *Bacillales* (15.6%, *n* = 18), and *Thermoanaerobiales* (8.7%, *n* = 10). The most represented families were *Peptococcaceae* (28.7%, *n* = 33), *Clostridiaceae* (10.4%, *n* = 12), *Bacillaceae* (11.3%, *n* = 13), and *Thermoanaerobacteraceae* (7%, *n* = 8) (Fig. 3; Supplementary Table 3). The number of detected thermospore OTUs per location ranged from 0 to 30 (on average, 9.8 ± 5.9 (sd), *n* = 111) (Supplementary Table S1). On average thermospore OTU richness was higher in Mississippi Canyon (14.7 ± 8.2 per location) and Abyssal Plain (10.2 ± 5.7 per location) sediments, compared to those in De Soto Valley (8.8 ± 4.6 per location) and West Florida Shelf (5.1 ± 2 per location) sediments, as depicted in Fig. 1.

### Distributions of thermophilic endospores in different geologic provinces of the Eastern Gulf of Mexico

The vast majority of the thermospore OTUs were either site-specific (observed in only one location) or regionally endemic (restricted within a particular geologic province); e.g., 87 OTUs were found in ten or fewer locations and 26 of these were specific to a single location (16 in oil-positive and 10 in oil-negative locations) (Fig. 3a; Supplementary Table S3). Several of these OTUs have not been reported in previous studies of thermospores in cold marine sediments from around the world.

The top 10 most widely distributed or ‘cosmopolitan’ OTUs were all detected at more than 30 locations across different geologic provinces within the study area (Fig. 3a). Bipartite networks built from the OTU presence–absence matrix revealed that these OTUs occurred in both oil-positive and oil-negative locations (Fig. 3b, c). The more clustered appearance of the oil-positive locations around the cosmopolitan OTUs in the first network (Fig. 3b) compared to a relatively distributed appearance of the oil-negative locations in the second network (Fig. 3c) suggests that the cosmopolitan OTUs co-occur at a greater frequency in the oil-positive locations. The cosmopolitan thermospore OTUs were related to *Caloranaerobacter* (OTU8), *Desulfotomaculum* (OTU2, OTU3, OTU10), *Gelria* (OTU7), *Moorella* (OTU5), *Sulfobacillus* (OTU1), *Symbiobacterium* (OTU9), and *Thermicanus* (OTU4), and in one case was only assigned at the family level (*Clostridiaceae*-4: OTU6) due to a lack of close, cultivated relatives.

### Certain thermospores are associated with migration of thermogenic hydrocarbons

**Fig. 4:**
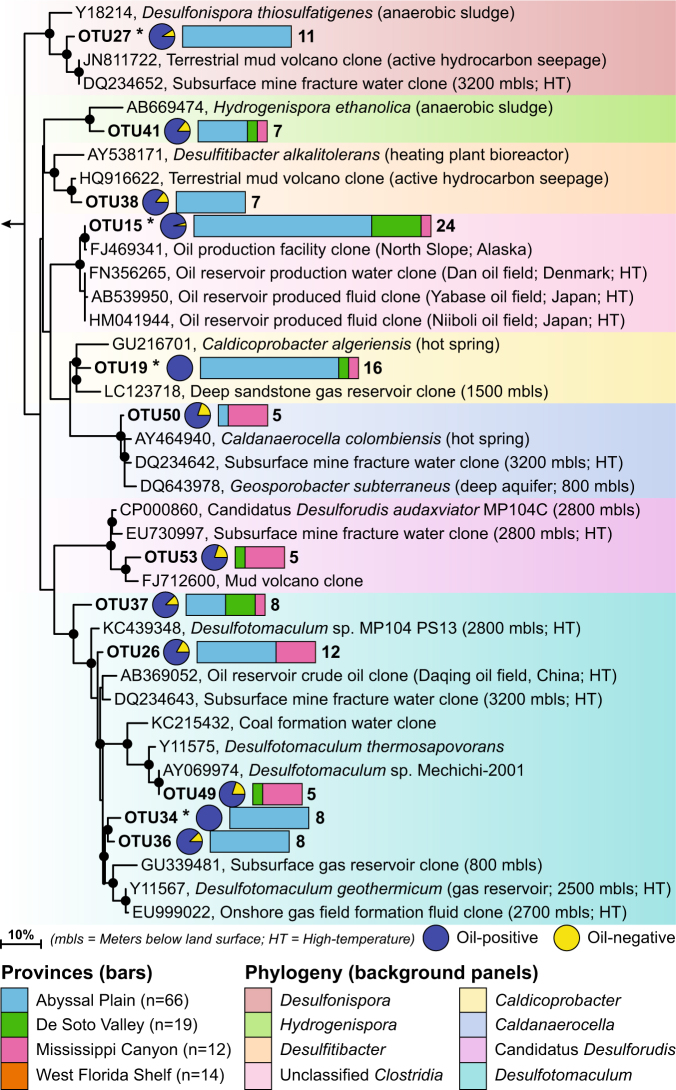
An annotated 16S rRNA-based phylogenetic tree showing 12 thermophilic endospore OTUs which represent eight lineages (highlighted in eight different background colors) within the anaerobic *Clostridiales* in the Phylum *Firmicutes*. Asterisks denote the four OTUs that are significantly correlated with migrated hydrocarbons. Two-colored pie charts indicate the percentage site occupancy for each of these OTUs in oil-positive and oil-negative ranked locations (cf. Fig. 2). Multi-colored stacked bars indicate site occupancies in different geologic provinces within the study area (none of these 12 OTUs were present in the West Florida Shelf province). The scale bar indicates 10% sequence divergence as inferred from PhyML. The black circles at the nodes of the branches indicate >80% bootstrap support (100 re-samplings). An extended tree showing the phylogeny and distribution of all 115 thermospore OTUs is presented in Supplementary Figure S5

**Fig. 5:**
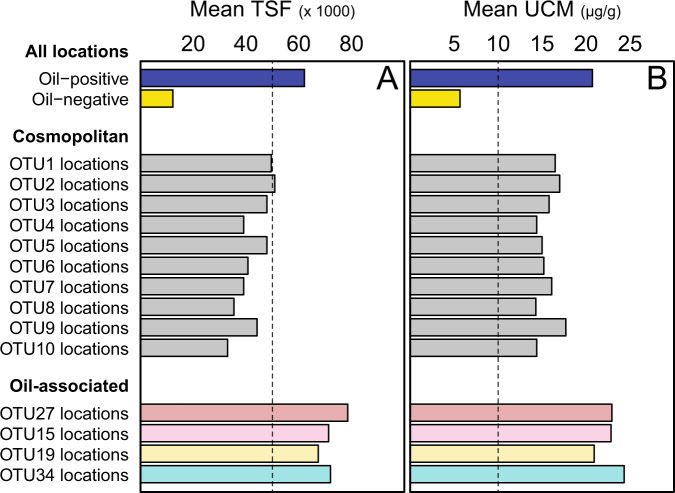
Mean values of TSF maximum intensity (**a**) and the UCM in µg/g dry sediment (**b**) for all oil-positive and oil-negative locations, for all locations where the top 10 cosmopolitan OTUs were observed (cf. Fig. 3), and for all locations where the 4 most strongly oil-associated OTUs were observed. Locations of the oil-associated OTUs are colored to match the respective OTU lineages indicated in the phylogenetic tree (cf. Fig. 4). Vertical dashed lines represent the threshold TSF intensity (50,000) and UCM (10 µg/g dry sediment) values used for classifying samples into oil-positive and oil-negative categories (cf. Fig. 2)

To highlight thermospore OTUs strongly associated with oil-positive locations, the OTU presence–absence matrix was screened for thermospores that were present in at least five locations overall, with at least 80% of those locations being oil-positive. This approach revealed 12 OTUs within eight thermospore lineages that occurred predominantly in oil-positive locations. All 12 were distributed only within the deeper and more western geologic provinces, and not detected in sediments in the shallower West Florida Shelf (Fig. 4; Supplementary Figure S5). Assessment of OTU occurrence with TSF and UCM values using point biserial correlation highlighted four of these OTUs (15, 19, 27, and 34) as having the strongest correlation (*p* < 0.05) with migrated hydrocarbons. As expected, these four OTUs were all found in locations with TSF and UCM values well above the average both for all oil-positive locations, and locations where the top 10 cosmopolitan OTUs were found (Fig. 5).

Most prominent among these was OTU15 (*r*_pb-TSF_ = 0.39; *r*_pb-UCM_ = 0.45; *p* < 0.001), detected in 24 locations (average water depth = 2743 m), 23 of which were oil-positive. This OTU can only be classified to the class level and BLAST searching using a representative sequence for this OTU showed fully identical top hits derived from only oil reservoirs and petroleum pipeline systems (data not shown). OTU19 (*r*_pb-TSF_ = 0.31; *r*_pb-UCM_ = 0.27; *p* < 0.001) belonging to the *Caldicoprobacter* clade, showed 100% site occupancy (*n* = 16; average water depth = 2866 m) in oil-positive sediments. Neither OTU15 nor OTU19 was confined to any given region within the EGoM study area, but most of their occurrences were in sediments in very deep water in the Abyssal Plain province (18 out of 24 for OTU15, and 14 out of 16 for OTU19).

Oil-associated OTU34 (*r*_pb-TSF_ = 0.21; *r*_pb-UCM_ = 0.29; *p* < 0.05) along with four other OTUs were affiliated with the sulfate-reducing *Desulfotomaculum* lineage. The occurrence of two of these five OTUs (OTUs 34 and 36) was confined to the deep water Abyssal Plain province (Fig. 4; average water depths = 3124 and 2961 m, respectively). One of the closest cultured relatives (96% sequence identity) to these two deep water *Desulfotomaculum* thermospores is *Desulfotomaculum* *geothermicum*, a sulfate-reducing bacterium first isolated from the production well of a saline, geothermally active underground gas reservoir in the Paris Basin [57]. *D*. *geothermicum* strains have also been isolated from petroleum-bearing deep subsurface settings such as sandstone aquifers in Eastern Germany [58], fracture water from South African gold mines [59, 60], and offshore oil reservoirs [27]. Closest uncultured relatives of the other three OTUs inhabit hot subsurface gas fields [61, 62] and crude oil from high-temperature oil reservoirs [63].

The *Desulfonispora* clade contained another oil-associated OTU (OTU27; *r*_pb-TSF_ = 0.19; *r*_pb-UCM_ = 0.28; *p* < 0.05), which remained confined within the deep water Abyssal Plain province along with OTU38, belonging to the *Desulfitibacter* clade. Both OTUs were close relatives to organisms found in a terrestrial mud volcano actively seeping petroleum hydrocarbons [64]. OTU50 and OTU53, belonging to *Caldanaerocella* and candidatus *Desulforudis* clades, respectively, were predominantly found in the Mississippi Canyon region. OTU41, belonging to the *Hydrogenispora* lineage, was detected in both deep and shallow sediments.

## Discussion

Multiple warm habitats including geological features such as deep petroleum reservoirs and fractured ocean crust [19] have been proposed as the source environments that could supply dormant endospores of thermophilic bacteria to permanently cold marine sediments. Terrestrial habitats associated with industry, e.g., anaerobic digesters and wastewater treatment plants, have also been postulated as sources [25, 43]. The large number of thermospore OTUs uncovered in this study (>100) and previous biogeography surveys [20] suggest that multiple dispersal vectors originating from different warm environments could be responsible for the diversity of thermospores observed in the world’s oceans. Focusing on petroleum-rich deep water Gulf of Mexico sediments provides an opportunity to better constrain and assess subsurface petroleum reservoirs as habitats of origin for thermophilic spore-forming bacteria in the marine environment. The dispersal history in this scenario requires expulsion from subsurface oil reservoirs into the overlying water column via hydrocarbon seepage, followed by deposition via sedimentation into the seabed, where thermospores can be detected using the methods employed here.

Restricted site occupancy demonstrated by a large proportion of the thermospore OTUs in our study (Fig. 3b, c) suggests a deterministic pattern of thermospore distribution within the EGoM area, and can be explained by local warm environments accompanied by dispersal limitation. Four novel thermospores with OTU occurrences significantly correlated with thermogenic hydrocarbons, and closest relatives from petroleum reservoirs, indicate that these four bacteria originated within petroleum-bearing subsurface sediments in the study area. For example, OTU15 showed the strongest correlation with migrated petroleum and its consensus sequence was identical (100% 16S rRNA gene sequence identity; Fig. 4) to sequences of uncultured *Clostridia* reported from four high-temperature oil reservoirs (Alaska’s North Slope, Dan in the Danish sector of the North Sea, and Yabase and Niiboli in Japan) around the world. This is the first time this lineage has been reported from a different environment other than a subsurface oil reservoir. The average depth and in situ temperatures of these oil fields range from 1100 to 2000 m and 40 to 80 °C ([27, 65–67], respectively). Of the 24 EGoM sediments where OTU15 was detected, 23 had liquid hydrocarbons well above the overall average for the 111 sediments in our study (Fig. 5). Similarly OTU19 was found exclusively in sediments with oil values (*n* = 16) at levels well above the average (Fig. 5). Its closest relative, within the genus *Caldicoprobacter* comes from a warm (40-45 °C) offshore petroleum reservoir (1500 m deep sandstone formation) harboring an indigenous microbial community dominated by *Clostridiales* [68].

The largest clade of thermospores (five OTUs including oil-associated OTU34) that were prevalent in the oil-positive locations belongs to the thermophilic *Desulfotomaculum*. Sulfate-reducing bacteria belonging to this genus have been isolated from oil reservoirs from around the world [69, 70]. *Desulfotomaculum* spp. are also well known inhabitants of other warm anoxic habitats, e.g., at mid-ocean ridges and in geothermally active aquifers [59]. In this regard, it should be noted that one of the most widely detected and cosmopolitan thermospore in these EGoM sediments (OTU2, found in 73 locations) also belongs to this genus. Thus, whereas *Desulfotomaculum* OTUs 26, 34, 36, 37, and 49 (Fig. 4) are most likely coming from EGoM oil reservoirs, the same cannot necessarily be concluded for the cosmopolitan *D. geothermicum* OTU2. Unlike the deep sea oil-associated thermospores, OTU2 was found in all four geologic provinces and in 18 out of the 40 oil-negative sediments (Fig. 3c). Either this particular *Desulfotomaculum* OTU is coming from a source environment that influences a larger area of the EGoM basin, or it is indeed also derived from subsurface petroleum reservoirs in our study area, but is more abundant or better equipped for widespread dispersal as has been shown for other closely related *Desulfotomaculum* spp. that produce extremely heat-resistant endospores that seem very well suited for hot, harsh subsurface conditions [71].

The identification of only 12 OTUs as prime candidates for having experienced vertical emigration with oil implies that a large majority of the thermospores detected in this study (i.e., >100) may not originate in EGoM oil reservoirs and could have different dispersal histories. For example, the large variation in site occupancy among the 30 OTUs within the *Desulfotomaculum* lineage (Supplementary Figure S5) suggests that their distribution is likely influenced by a variety of dispersal vectors. Ocean currents have been argued to play a key role in passive dispersal of thermospores to distant locations from their point of origin [20]. Water mass circulation features in the semi-enclosed GoM basin are predominantly influenced by the Loop Current, a warm surface current [72] affecting this study area (Fig. 1; inset map). Passive dispersal of the more cosmopolitan OTUs from one geologic province to another, or more broadly, immigration into the EGoM from distant sources, e.g., the hydrocarbon seeps in the Caribbean Sea [30], via the Loop Current, may partly explain their widespread occurrence in the study area. Conversely, some of the locally restricted OTUs could also emigrate out of the EGoM through the Loop Current.

Multiple dispersal histories among the vast majority of thermospore OTUs underscore the uniqueness of the few OTUs that are likely dispersed via a deep-to-shallow mechanism. The 12 oil-associated *Clostridia* represent new candidate bioindicators for investigating hydrocarbon seepage as a dispersal vector connecting the deep and shallow biospheres. The reservoirs underneath the deep water basin of the Gulf of Mexico remain relatively underexplored compared to the well known working petroleum systems in its shallower north-western and north-central parts [73]. According to the geothermal gradient in the deep EGoM, this region is expected to contain interbedded sandstone and shale reservoirs with predicted formation temperatures ranging from 30 to 100 °C [74] consistent with the physiology observed for the thermophilic bacteria discovered in this study, and inferred for their close relatives from other oil reservoirs around the world. These thermospores may therefore be useful for understanding deep oil reservoir microbiology and biogeochemistry in this region.

The oil reservoir microbiome examples highlighted above along with several others indicate that spore-forming *Firmicutes* are commonly found in these environments, especially deeper reservoirs with temperatures above 50 °C [40]. Besides these *Clostridia*, several other lineages of bacteria and archaea also inhabit oil reservoirs. Certain *Thermotogae* have only ever been found in oil reservoirs [75–77], an endemism suggesting that in principle they could also be cold sediment bioindicators for deeper reservoir environments if they are similarly ejected via hydrocarbon seeps and can in future also be detected at the seabed [13, 78, 79]. While it is not necessary that only spore-forming thermophiles would be subject to warm-to-cold upward transmigration, the results presented here reveal the ease with which thermospores can be studied as fingerprints of deep life and showcase petroleum geofluids as dispersal vectors connecting the deep and shallow biospheres.

## Electronic supplementary material

Supplementary Information(DOCX 25 kb)

Supplementary Figure S1(PDF 262 kb)

Supplementary Figure S2(PDF 226 kb)

Supplementary Figure S3(PDF 326 kb)

Supplementary Figure S4(PDF 317 kb)

Supplementary Figure S5(PDF 175 kb)

Supplementary Table S1(XLSX 64 kb)

Supplementary Table S2(XLSX 53 kb)

Supplementary Table S3(XLSX 111 kb)
