# Supplementary material for: Thermophilic endospores associated with migrated thermogenic hydrocarbons in deep Gulf of Mexico marine sediments
Source: ISME J. 2018 Mar 29;12(8):1895–906. doi: 10.1038/s41396-018-0108-y (PMC6052102; doi:10.1038/s41396-018-0108-y)
Supplement: Supplementary file 1 — Supplementary Information(DOCX 25 kb) [file 41396_2018_108_MOESM1_ESM.docx]

**Supplementary Methods**

**Sulfate and organic acid concentrations**

Supernatants from slurry sub-samples were filtered through 0.22 µm pore-size syringe filters (13 mm diameter) to remove any remaining suspended particles. Sulfate concentrations were measured in a Dionex ICS-5000 reagent-free ion chromatography system (Thermo Scientific, CA, USA) equipped with an anion-exchange column (Dionex IonPac AS22; 4 x 250 mm; Thermo Scientific), an EGC-500 K_2_CO_3_ eluent generator cartridge and a conductivity detector. Concentrations of low-molecular-weight organic acids were determined using reverse-phase separation followed by UV detection (210 nm) on a Dionex Ultimate-3000 HPLC system (Thermo Scientific) equipped with an Acclaim Organic Acid column (5 µm; 4 x 250 mm; Thermo Scientific). The eluent was 100 mM Na_2_SO_4_ prepared using HPLC grade water and pH corrected to 2.65 using methanesulfonic acid.

**16S rRNA gene amplicon sequencing**

Each PCR reaction consisted of 1 μL (~20 ng) genomic DNA template, 2.5 μL of each of the primers (final concentration 1 μM), 12.5 μL 2X Kapa HiFi HotStart ReadyMix (Kapa Biosystems, Wilmington, MA, USA) and sterile nuclease free water to make a final volume of 25 μL. In order to achieve optimal annealing, a touchdown PCR program was designed, as follows: initial denaturation at 95°C for 5 min, 10 cycles of 95°C for 30 sec, 60°C (-1°C /cycle) for 45 sec, 72°C for 1 min, followed by 20 cycles of 95°C for 30 sec, 55°C for 45 sec, 72°C for 1 min, and final extension at 72°C for 5 min. All PCR reactions were performed in triplicate, pooled, and purified using the NucleoMag NGS Clean-up and Size Select kit (Macherey-Nagel Inc., Bethlehem, PA, USA). The purified PCR products were indexed following the instructions on Illumina’s 16S amplicon library preparation guide. The concentration of dsDNA and the size of the indexed amplicons were verified using the Qubit dsDNA High Sensitivity assay kit on a Qubit 2.0 fluorometer (Thermo Fisher Scientific, Canada) and the High Sensitivity DNA kit on an Agilent 2100 Bioanalyzer system (Agilent Technologies, Mississauga, ON, Canada), respectively.

**Supplementary Results**

**Hydrocarbon analyses**

Additional hydrocarbon characterization other than TSF and UCM included measurement of total C_15+_ alkanes which showed a distribution of 0.2-9.3 µg/g dry sediment (Supplementary Figure S1). Thermogenic alkanes were defined as the fraction of the total C_15+_ alkanes excluding odd-numbered C_27-33_ alkanes (owing to these being diagenetic plant-derived waxes and indicators of recent organic matter). The thermogenic/diagenetic (T/D) ratio reflected the ratio of thermogenic C_15+_ alkanes to C_27-33­_ alkanes. T/D ratios ranged from 0.7-4.1 among all locations and showed positive correlation with the total C_15+_ alkanes (*R^2^*=0.386; n=111; Supplementary Figure S1), which suggested that thermogenicity increased with increasing alkane concentrations.

**Incubation conditions**

In order to design a high-temperature incubation strategy to apply to all EGoM samples, we initially investigated the effects of incubation temperature, different electron donors, and slurry subsampling intervals on a smaller subset of sediments. A microcosm using pasteurized sediment from EGM035 was amended with the six organic acids mix, incubated at 50°C and subsampled seven times within the first 15 days. Another pasteurized sample (EGM076) was used to prepare microcosms that were separately amended with the six organic acids mix as well as each individual organic acid and was incubated for two weeks. While all incubations showed sulfate reduction and depletion of organic acids to various extents, thermospore OTU richness was greatest in subsamples collected on day 14 (EGM035) and in microcosms amended with the six organic acids mix, respectively (Supplementary Figure S2). The same experiments with both sediments were repeated at 70°C but no sulfate reduction or depletion of organic acids was observed after two weeks (data not shown). Based on these results, the experimental design with the amendment of the six organic acids mix and subsampling after 14 days at 50°C was chosen for the 111 sediments. The onset and extent of sulfate reduction, when observed, varied between the samples and also among replicates of the same sample (Supplementary Table S1). For eight of the sediment locations, the community composition among *Firmicutes* in amplicon libraries from individual replicates were compared with an amplicon library prepared from a pooled sample (i.e. combining equal volumes of subsamples from triplicate slurries prior to DNA extraction). This revealed that the families that became prominent in the individual replicate slurries from each location were also detected in the mixed slurry from the same location (Supplementary Figure S3).

**Supplementary figure and table legends**

**Supplementary Figure S1:** Scatterplot showing the mean values (n=3 extracts) of two additional geochemical parameters used for confirming the thermogenicity trend of the hydrocarbons in 111 sediment cores. The plot is accompanied by two marginal box-and-whisker plots summarizing the distribution (minimum, lower quartile, median, upper quartile and maximum) for each of the parameters on the corresponding parallel axes. The dashed line represents the linear regression of the parameters with the corresponding *R^2^* value indicated. Symbol colors indicate the number of oil-qualified extracts (n=0, 1, 2 or 3 out of 3) in each sample. Symbol shapes indicate the different geologic provinces.

**Supplementary Figure S2:** Bar plots showing impact of incubation conditions on number of detected thermospore OTUs. Panels A and B show differences in detection of thermospore OTUs when one sample (EGM035) amended with the six organic acids mix was subsampled at different intervals (days of incubation), and when another sample (EGM076) was incubated with and without amendment with various electron donors and subsampled at the same interval (after six days of incubation), respectively.

**Supplementary Figure S3:** Relative abundance of indicated families within the phylum *Firmicutes* in 14d amplicon libraries from eight sediment locations. Pooled triplicate slurries (left most bars) were used for DNA extraction and library preparation, and were compared with libraries derived from each of the triplicates individually (three right most bars). In all eight cases, all of the groups observed in the individual samples were also observed in the pooled sample.

**Supplementary Figure S4:** PCoA plot of weighted UniFrac distances (Panel A) and NMDS plot of Bray-Curtis distances (Panel B) showing the shift in bacterial community composition before (0d) and after (14d) high-temperature incubation of pasteurized marine sediments (n=111) for 14 days. All analyses were performed after rarefication of all libraries to 5000 sequences. Symbol colors represent 0d and 14d amplicon libraries and symbol shapes represent oil-negative and oil-positive categories, respectively.

**Supplementary Figure S5:** Annotated 16S rRNA gene based phylogenetic tree of 115 thermophilic endospore OTUs within the phylum *Firmicutes* detected in EGoM sediments. Scale bar indicates 10% sequence divergence as inferred from PhyML. Two-colored pie charts indicate degree of association (percentage site occupancy) each OTU has with oil-positive and oil-negative samples. Multi-colored stacked bars indicate the total site occupancy for each OTU, organized by color for the different geologic provinces within the study area. The background panels, when shown, indicate the clades representing the 12 thermospore OTUs preferentially occurring in oil-positive locations. The color of the background panels are the same as shown in Figure 4. The tree was constructed with bootstrap support (100 samplings) but the bootstrap values are not displayed. *Geobacter metallireducens* (accession number L07834) was used as outgroup during tree reconstruction (not shown).

**Supplementary Table S1:** Marine sediment sample description, thermospore OTU richness, and thermophilic sulfate reduction rates.

**Supplementary Table S2:** Read number, coverage, and alpha diversity of bacterial 16S rRNA gene amplicon libraries of marine sediments before and after pasteurization followed by incubation at 50°C.

**Supplementary Table S3:** Site occupancy, taxonomy, next relatives and presence/absence at sampling locations of thermospore OTUs.
