# Supplementary material for: Thermophilic endospores associated with migrated thermogenic hydrocarbons in deep Gulf of Mexico marine sediments
Source: ISME J. 2018 Mar 29;12(8):1895–906. doi: 10.1038/s41396-018-0108-y (PMC6052102; doi:10.1038/s41396-018-0108-y)
Supplement: Supplementary file 2 — Supplementary Figure S1(PDF 262 kb) [file 41396_2018_108_MOESM2_ESM.pdf]

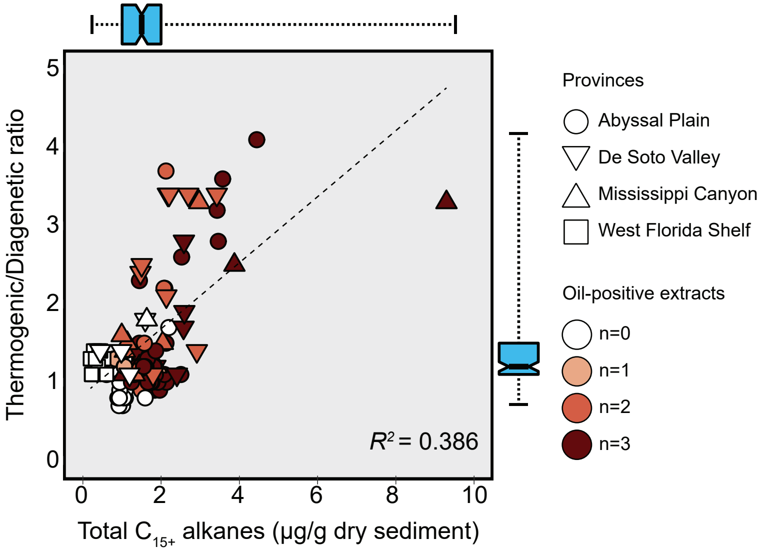

Supplementary Figure S1: Scatterplot showing the mean values (n=3 extracts) of two additional geochemical parameters used for confirming the thermogenicity trend of the hydrocarbons in 111 sediment cores. The plot is accompanied by two marginal box-and-whisker plots summarizing the distribution (minimum, lower quartile, median, upper quartile and maximum) for each of the parameters on the corresponding parallel axes. The dashed line represents the linear regression of the parameters with the corresponding R^2 value indicated. Symbol colors indicate the number of oil-qualified extracts (n=0, 1, 2 or 3 out of 3) in each sample. Symbol shapes indicate the different geologic provinces.
