# Supplementary material for: Thermophilic endospores associated with migrated thermogenic hydrocarbons in deep Gulf of Mexico marine sediments
Source: ISME J. 2018 Mar 29;12(8):1895–906. doi: 10.1038/s41396-018-0108-y (PMC6052102; doi:10.1038/s41396-018-0108-y)
Supplement: Supplementary file 3 — Supplementary Figure S2(PDF 226 kb) [file 41396_2018_108_MOESM3_ESM.pdf]

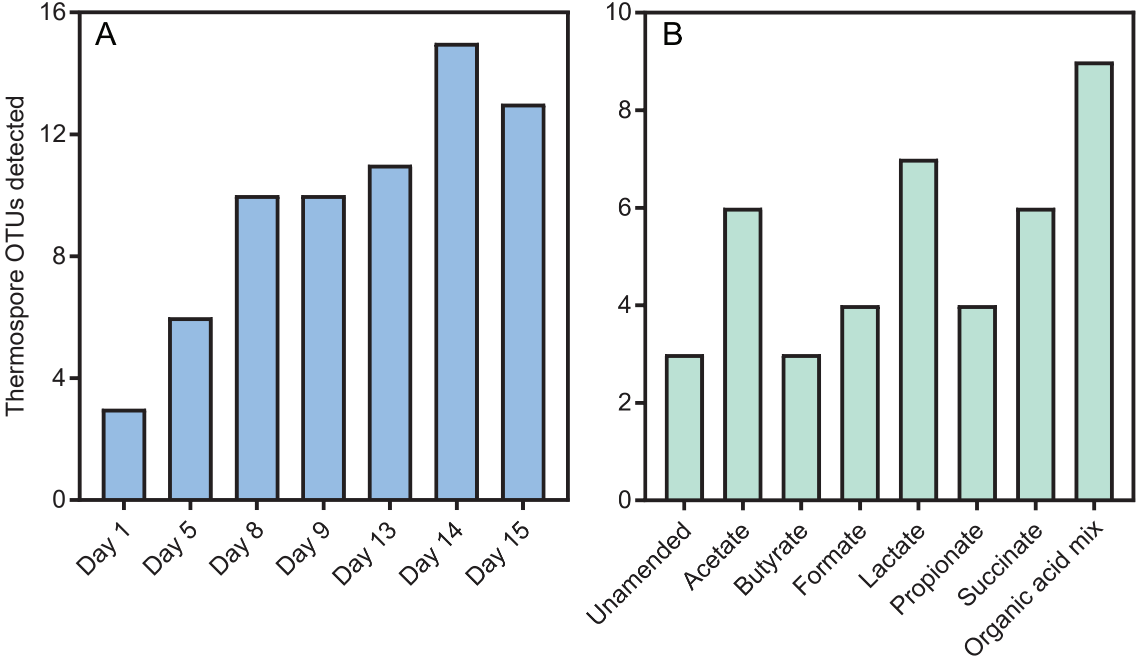

Supplementary Figure S2: Bar plots showing impact of incubation conditions on number of detected thermospore OTUs. Panels A and B show differences in detection of thermospore OTUs when one sample (EGM035) amended with the six organic acids mix was subsampled at different intervals (days of incubation), and when another sample (EGM076) was incubated with and without amendment with various electron donors and subsampled at the same interval (after six days of incubation), respectively.
