# Supplementary material for: Thermophilic endospores associated with migrated thermogenic hydrocarbons in deep Gulf of Mexico marine sediments
Source: ISME J. 2018 Mar 29;12(8):1895–906. doi: 10.1038/s41396-018-0108-y (PMC6052102; doi:10.1038/s41396-018-0108-y)
Supplement: Supplementary file 4 — Supplementary Figure S3(PDF 326 kb) [file 41396_2018_108_MOESM4_ESM.pdf]

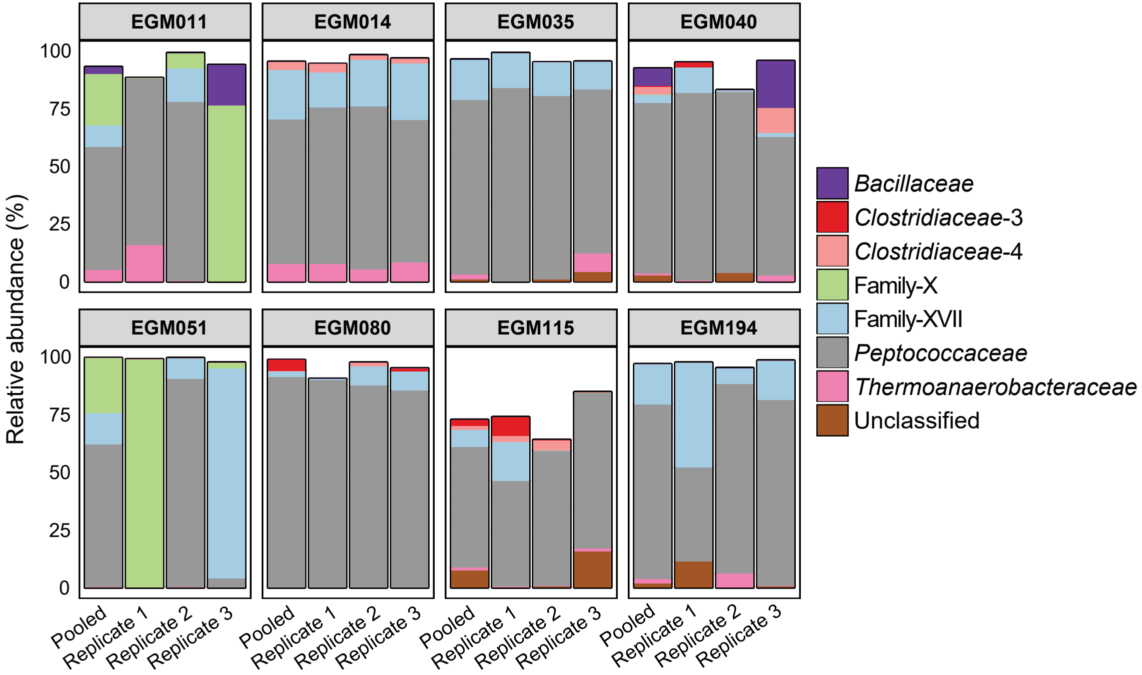

Supplementary Figure S3: Relative abundance of indicated families within the phylum *Firmicutes* in 14d amplicon libraries from eight sediment locations. Pooled triplicate slurries (left most bars) were used for DNA extraction and library preparation, and were compared with libraries derived from each of the triplicates individually (three right most bars). In all eight cases, all of the groups observed in the individual samples were also observed in the pooled sample.
