# Supplementary material for: Thermophilic endospores associated with migrated thermogenic hydrocarbons in deep Gulf of Mexico marine sediments
Source: ISME J. 2018 Mar 29;12(8):1895–906. doi: 10.1038/s41396-018-0108-y (PMC6052102; doi:10.1038/s41396-018-0108-y)
Supplement: Supplementary file 5 — Supplementary Figure S4(PDF 317 kb) [file 41396_2018_108_MOESM5_ESM.pdf]

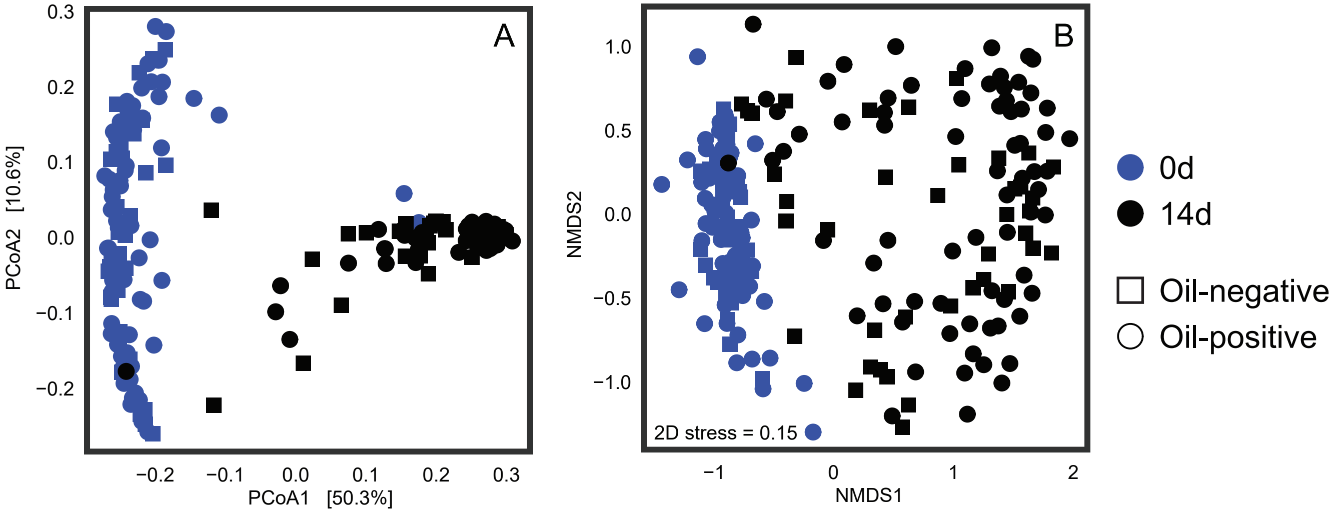

Supplementary Figure S4: PCoA plot of weighted UniFrac distances (Panel A) and NMDS plot of Bray-Curtis distances (Panel B) showing the shift in bacterial community composition before (0d) and after (14d) high-temperature incubation of pasteurized marine sediments (n=111) for 14 days. All analyses were performed after rarefication of all libraries to 5000 sequences. Symbol colors represent 0d and 14d amplicon libraries and symbol shapes represent oil-negative and oil-positive categories, respectively.
