# Supplementary material for: Thermophilic endospores associated with migrated thermogenic hydrocarbons in deep Gulf of Mexico marine sediments
Source: ISME J. 2018 Mar 29;12(8):1895–906. doi: 10.1038/s41396-018-0108-y (PMC6052102; doi:10.1038/s41396-018-0108-y)
Supplement: Supplementary file 6 — Supplementary Figure S5(PDF 175 kb) [file 41396_2018_108_MOESM6_ESM.pdf]

Supplementary Figure S5
(1/3)

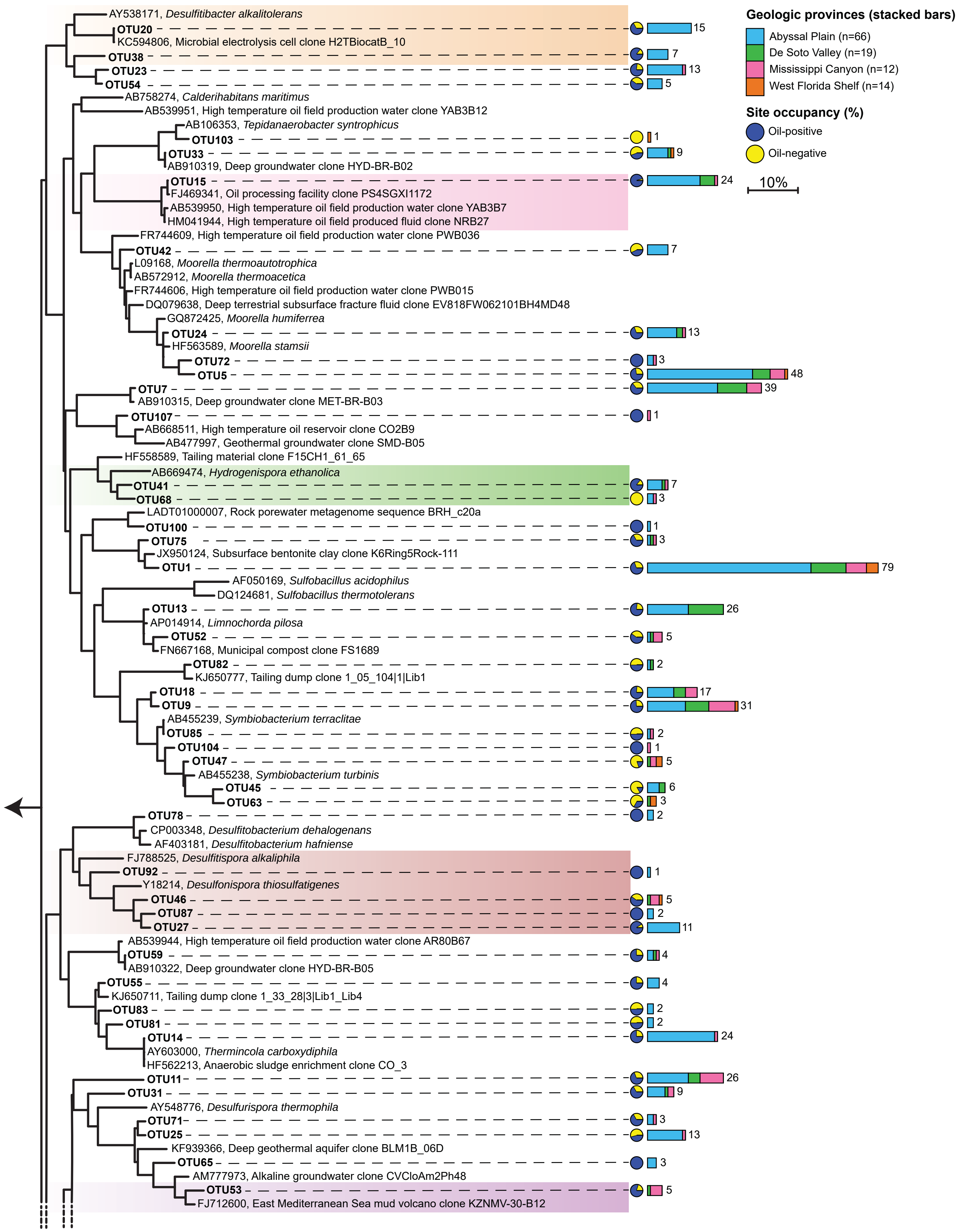

Supplementary Figure S5
(2/3)

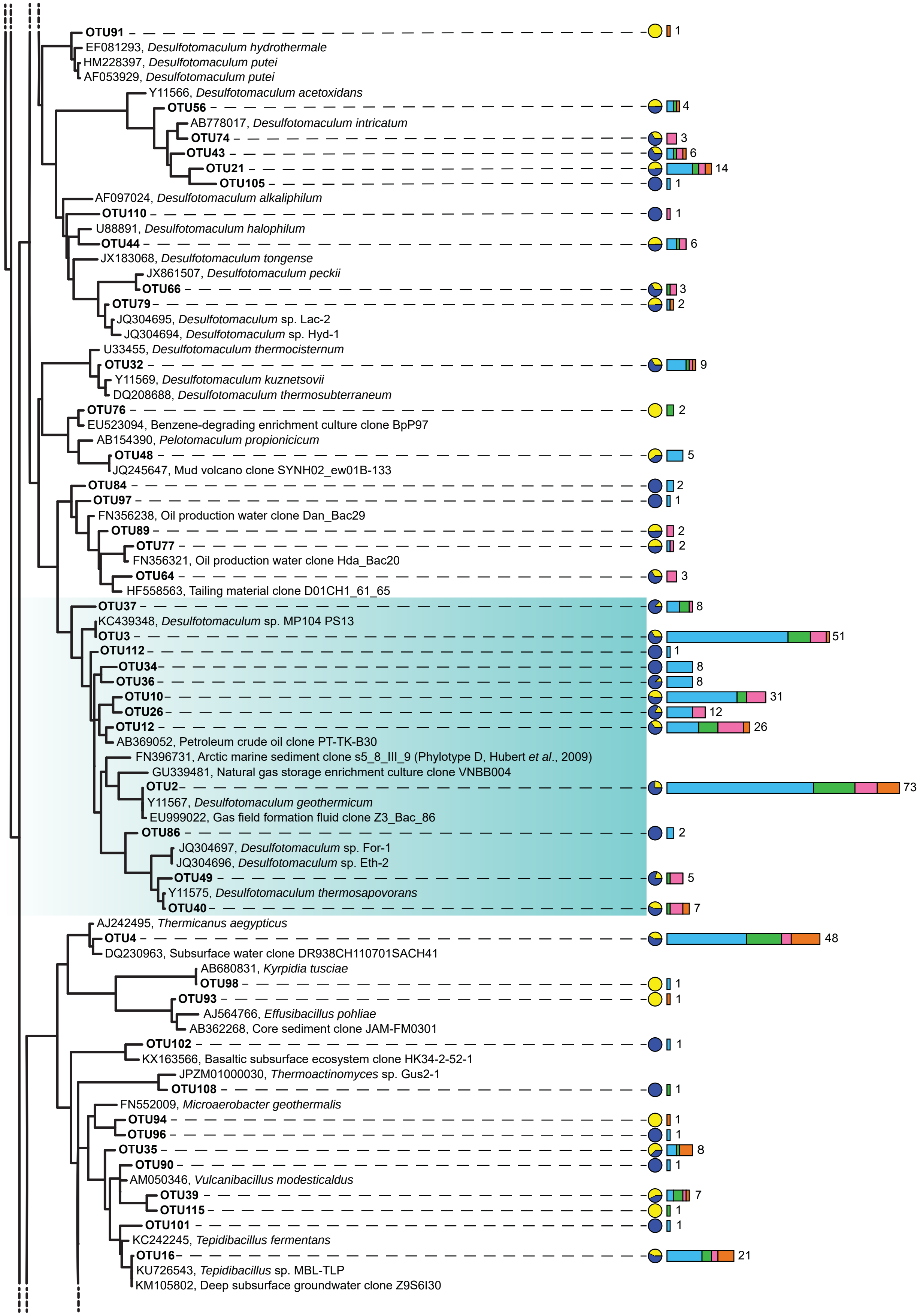

Supplementary Figure S5
(3/3)

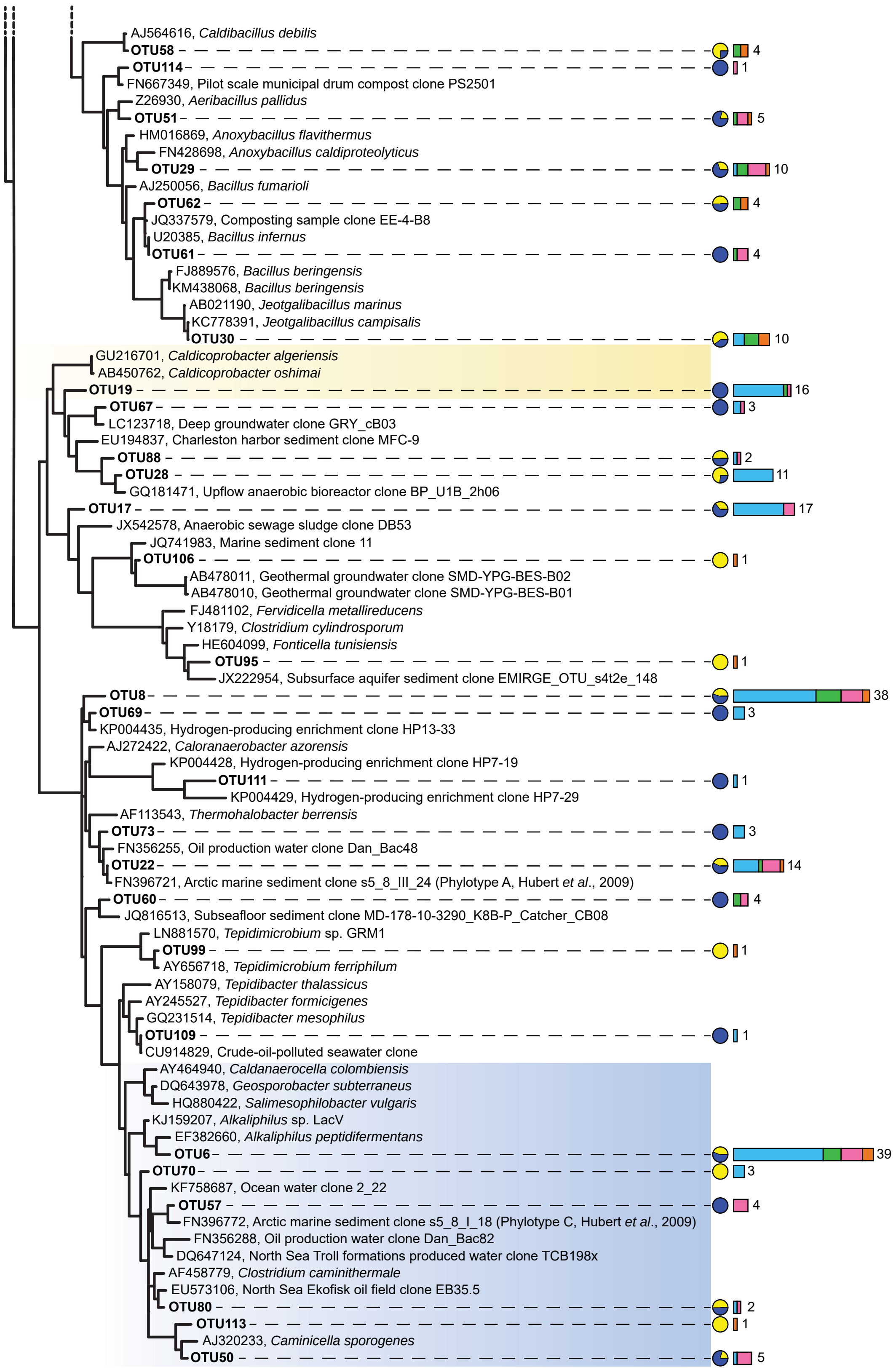

Supplementary Figure S5: Annotated 16S rRNA gene based phylogenetic tree of 115 thermophilic endospore OTUs within the phylum *Firmicutes* detected in EGoM sediments. Scale bar indicates 10% sequence divergence as inferred from PhyML. Two-colored pie charts indicate degree of association (percentage site occupancy) each OTU has with oil-positive and oil-negative samples. Multi-colored stacked bars indicate the total site occupancy for each OTU, organized by color for the different geologic provinces within the study area. The background panels, when shown, indicate the clades representing the 12 thermospore OTUs preferentially occurring in oil-positive locations. The color of the background panels are the same as shown in Figure 4. The tree was constructed with bootstrap support (100 samplings) but the bootstrap values are not displayed. *Geobacter metallireducens* (accession number L07834) was used as outgroup during tree reconstruction (not shown).
